# The decrease in silicon concentration of the connective tissues with age in rats is a marker of connective tissue turnover^☆^

**DOI:** 10.1016/j.bone.2015.02.004

**Published:** 2015-06

**Authors:** Ravin Jugdaohsingh, Abigail I.E. Watson, Liliana D. Pedro, Jonathan J. Powell

**Affiliations:** a MRC Human Nutrition Research, Elsie Widdowson Laboratory, Cambridge, United Kingdom; b School of Sport and Exercise Health Sciences, Loughborough University, Loughborough LE11 3TU, UK

**Keywords:** Collagen, Connective tissues, Growth & development, Whole body Si content, Silicon status, Silicon requirement

## Abstract

Silicon may be important for bone and connective tissue health. Higher concentrations of silicon are suggested to be associated with bone and the connective tissues, compared with the non-connective soft tissues. Moreover, in connective tissues it has been suggested that silicon levels may decrease with age based upon analyses of human aorta. These claims, however, have not been tested under controlled conditions. Here connective and non-connective tissues were collected and analysed for silicon levels from female Sprague–Dawley rats of different ages (namely, 3, 5, 8, 12, 26 and 43 weeks; n = 8–10 per age group), all maintained on the same feed source and drinking water, and kept in the same environment from weaning to adulthood. Tissues (696 samples) were digested in nitric acid and analysed by inductively coupled plasma optical emission spectrometry for total silicon content. Fasting serum samples were also collected, diluted and analysed for silicon. Higher concentrations of silicon (up to 50-fold) were found associated with bone and the connective tissues compared with the non-connective tissues. Although *total* silicon content increased with age in all tissues, the highest connective tissue silicon *concentrations* (up to 9.98 μg/g wet weight) were found in young weanling rats, decreasing thereafter with age (by 2–6 fold). Fasting serum silicon concentrations reflected the pattern of connective tissue silicon concentrations and, both measures, when compared to collagen data from a prior experiment in Sprague–Dawley rats, mirrored type I collagen turnover with age. Our findings confirm the link between silicon and connective tissues and would imply that young growing rats have proportionally higher requirements for dietary silicon than mature adults, for bone and connective tissue development, although this was not formally investigated here. However, estimation of total body silicon content suggested that actual Si requirements may be substantially lower than previously estimated which could explain why absolute silicon deficiency is difficult to achieve but, when it is achieved in young growing animals, it results in stunted growth and abnormal development of bone and other connective tissues.

## Highlights

• Silicon may be important for bone and connective tissue health.
• Higher silicon concentrations are found in connective tissues.
• Highest concentrations are found in young weanling rats and decreases with age.
• Young growing rats may have higher requirements for silicon than adults.
• Total body silicon content suggests requirement is lower than previously estimated.

## Introduction

Silicon (Si) is a natural trace element of the mammalian diet and although it has not been demonstrated *unequivocally* that mammals have a requirement for Si [1,2] there is increasing evidence to suggest that it may be important for the normal health of bone and the connective tissues [2,3]. Indeed, severe dietary Si deprivation in growing animals appears to cause abnormal growth and defects of the connective tissues [4,5]. Whilst these dramatic effects have not been replicated in the more recent studies, some effects on bone have been reported [6–15]. Moreover, Jugdaohsingh et al. [16] reported that rats on a Si-deprived diet actively conserve Si by reabsorption of urinary Si to maintain tissue Si levels. Silicon responsive genes have been identified from the kidneys of the Si-deprived rats [17]. In humans, higher dietary Si intakes are associated with optimal bone health and this appears to be mediated by oestrogen status [18,19]. The exact role of Si, however, is not known. A number of potential roles have been suggested including an involvement in DNA synthesis and in increasing/improving the utilisation of essential elements involved in bone and connective tissue health [20]. Silicon has a unique affinity for aluminium (Al), preventing its absorption in animals and humans [21,22] and it has also been proposed that it may bind and sequester toxic Al ions that may have detrimental effects on bone health [23,24].

However, much of the evidence points to an direct involvement of Si in extracellular matrix synthesis and/or its stabilisation, particularly so for collagen which is one of the main structural proteins of bone and connective tissues [3,24]. Higher levels of Si are reported to be associated with healthy connective tissues (e.g. aorta, bone, trachea, tendon etc.), and especially their connective tissue components (i.e. collagen and elastin), compared to non-connective tissues (e.g. liver, kidney, spleen etc.) [25–27]. Work by Alder and Berlyne using 31-labelled Si similarly showed that although much (~ 80%) of the Si was excreted in urine following injection, some was retained by the tissues, with high levels in skin and bone [28,29].

The concentration of Si in connective tissues is suggested to decrease with age and with disease progression, based upon data for the human aorta [27,30]. Fasting serum Si concentrations may also decrease with age, with higher concentrations in newborns, infants, toddlers and young children and lower concentrations in adolescents and adults [31,32]. Fasting serum Si concentrations could, therefore, be an indicator of connective tissue Si levels. However, these putative associations, namely (i) silicon's change in concentration with age in connective tissues, (ii) the change in fasting serum Si concentration with age and (iii) that the latter may reflect connective tissue levels, have not been assessed in a formal, controlled longitudinal setting. Indeed, whether Si definitely targets connective tissues, in preference to the major soft tissues/organs that often accumulate trace minerals (e.g. liver, lung and kidney), has not been assessed with modern analytical techniques.

In this work we have revisited the question of the levels of Si in the connective tissues (aorta, bone, trachea, etc.) versus non-connective tissues (kidney and liver) in a prospective controlled study and used modern, robust analytical methods. In addition, we have investigated whether levels of Si in the connective (and non-connective tissues) do indeed change with age, between weaning and adulthood, and seen how this compares to fasting serum Si levels. Finally, comparison was made between serum Si level's and a serum marker of type I collagen turnover, namely serum carboxy terminal telopeptide of type I collagen (CTx) to determine whether fasting serum Si is an indicator of collagen (connective tissue) turnover.

Note, this study did not set out to measure the distribution of Si in the rat's tissues, hence some tissues including brain, muscle and adipose tissue were not collected and analysed for Si. Previous analysis, however, showed that these tissues are unlikely to contain high levels of Si [25,29].

## Materials and methods

### Materials

Needles and syringes were from Terumno (Terumno Europe NV). Polypropylene sample collection tubes (13 mL, 25 mL & 50 mL) were from Sarstedt Ltd (Leicester, UK). Ultra high purity (UHP) water was 18 MΩ/cm, from a Branstead Nano-Pure water purifier (Thermo Scientific; Ohio, USA). Hydrogen peroxide (40%, AristaR grade) was from VWR International Ltd (Poole, UK). High purity nitric acid (69% (w/v) p.a. plus) was from Fluka Ltd (Gillingham, UK). Dialysis membranes (12.5–14 kDa) were from Spectrum Lab Inc (UK). ICP standard solutions, 1000 mg element/L, were from Merck Ltd (Poole, UK) or Sigma-Aldrich Chemical Co. (Gillingham, UK). A 100 mg/L multi-element standard, was prepared by pooling 1 mL aliquots of the 1000 mg/L ICP standard solution of the elements of interest.

### Rats

Female Sprague–Dawley rats of six different ages (Table 1) were from the National Heart and Lung Institute, Royal Brompton Hospital, London, UK. The rats were bred in-house and those sacrificed were ‘surplus to requirement’. Rats were maintained in plastic cages with stainless steel covers at 22 °C with 12 h/12 h light/dark cycle. All rats received (ad libitum) the same feed (SDS RM1 Expanded; Special Diets Service, UK) and drinking water throughout the 43 weeks. The silicon contents of the feed and drinking water were determined by analysis (see below).

### Tissue collection

Prior to sacrifice, rats were fasted overnight from 17.00 h: the feed was removed and only drinking water was allowed over this fasting period. At 09.00 h the following day, rats were killed by exposure to carbon dioxide gas (a Schedule 1 method of euthanasia approved by the Home Office Animals Scientific Procedures Act 1986; https://www.gov.uk/government/uploads/system/uploads/attachment_data/file/229022/0193.pdf), weighed and then immediately dissected. A blood sample was collected from the abdominal aorta, vena cava and the heart using a 2.5–10 mL syringe and 21 or 23G needle (depending on the age). The following tissues were then collected: heart, both lungs, a sample of the trachea, oesophagus and aorta, liver, both kidneys, sternum, right and left back limbs, both ear lobes and a sample of abdominal skin and fur. The spleen was also collected but was not analysed for all animals. All tissues were cleaned of adherent fat, washed thoroughly with high purity water, blotted dry and then placed into specimen tubes (25 mL polypropylene tube). The right and left back limbs were cleaned of excess flesh, tendons and ligaments and placed in 25 mL specimen tubes. At the end of each day's collection, all samples were couriered to MRC HNR on ice. At MRC HNR, the blood samples were stored at 4 °C overnight in 13 mL polypropylene tubes to clot and the following day were centrifuged at 3000 rpm (Sorvall RC3BP; Thermo Scientific, UK) for 10 min at 4 °C and the separated sera collected in to clean 13 mL polypropylene tubes and stored at − 80 °C. All other samples (tissues) were stored immediately at − 80 °C upon arrival at MRC HNR.

Extreme care was taken throughout the study, i.e. in the collection, preparation and analysis of the tissues, to avoid contamination of the samples with Si, as previously described [16].

### Total elemental analysis

Total elemental analysis for Si was carried out at 251.611 nm by inductively coupled plasma optical emission spectrometry (ICP-OES) using a Jobin Yvon 2000-2 (Instrument SA, Longjumeau, France) equipped with a concentric nebuliser and cyclonic spray chamber. Sample flow rate was 1 mL/min. Peak profiles were used as previously described [33,34], with a window size of 0.08 nm (0.04 nm either side of the peak) with 21 increments per profile and an integration time of 0.5 s per increment.

#### Serum samples

The serum samples were thawed at room temperature and an aliquot (0.2–1 mL) diluted 1 + 4 with 0.2% nitric acid (prepared by diluting 1.44 mL 69% nitric acid in 498.56 mL ultra-pure water). Two mL of diluted serum were removed from some of the samples and pooled together for the preparation of pooled sample-based standards: aliquots of the pooled, diluted serum were spiked with 0–100 μg/L Si from a 10 mg/L Si standard (prepared from the stock 1000 mg/L Si ICP solution). Silicon content of the diluent (0.2% nitric acid solution) was also determined using sample-based standards (0–100 μg/L) prepared in 0.2% nitric acid solution. The Si concentration of the diluent was subtracted from the Si concentration of the diluted samples, prior to correcting for dilution of the samples (× 5) to obtain the concentration in the original (undiluted) sera. Mean Si concentration of the diluent was 28.4 ± 0.4 μg/L (n = 3).

#### Soft tissues

Soft tissues (aorta (0.03–0.13 g), heart (0.24–0.98 g), oesophagus (0.03–0.10 g), trachea (0.05–0.43 g), lungs (0.73–1.8 g), sternum (0.02–0.08 g), ear lobes (0.17–0.37 g), kidney (0.28–0.91 g) and liver (1.8–2.6 g)) were thawed to room temperature, cleaned of adherent fat and hairs (i.e. for the ear lobes), rinsed thoroughly with UHP water to remove as much blood as possible, blotted dry and digested with 0.5–4.0 ml of an acid mixture containing equal volumes of concentrated (69%) nitric acid and 40% hydrogen peroxide in 13 or 25 mL polypropylene tubes. Sample blanks were similarly prepared, without sample (i.e. acid mixture alone). Samples and sample blanks were digested at 45–50 °C in a shaking incubator (HT Infors Minitron; Infors AG, Basel, Switzerland) set at 60–80 rpm. The whole of: lungs, heart, sternum, the pair of ear lobes and one kidney were digested from each rat. Except for 3-week old rats, where the whole liver was used per rat, one lobe of the liver was digested for each of the older rats. Samples required at least 72 h to completely digest and further addition (0.5–1 mL) of the acid mixture was required for some tissues. Regular venting of the sample tubes was also required to release any build-up of pressure. Upon complete digestion the samples and sample blanks were diluted 1 + 1 with UHP water. Aliquots of the diluted digested samples were removed and pooled together for the preparation of pooled sample-based standards (+ 0–2 mg Si/L) for each tissue type. Silicon levels in the sample blanks was determined with sample-based standards (+ 0–2 mg/L) prepared in the 1 + 1 diluted acid mixture. Each tissue type, except for trachea and oesophagus, was analysed as a single, separate batch consisting of all 58 digested samples, blanks and standards. Trachea digests and oesophagus digests were analysed in a single larger run (i.e. 116 digested samples, blanks and standards). Silicon concentration of the tissue samples was calculated using the method described below. Mean Si concentration of the sample blanks was 42.1 ± 2.5 μg/L (n = 31).

#### Bone samples

The right limbs were thawed to room temperature and further processed to remove any remaining flesh, tendons and ligaments. The tibia (0.10–0.66 g) and femur (0.13–0.85 g) were carefully separated and digested separately in 5–10 mL 10% nitric acid at 185 °C (10 min ramp to 185 °C and maintained at 185 °C for 20 min) in acid-cleaned polytetrafluoroethylene (PTFE, Teflon) vessels (made from second generation tetrafluoroethylene, TFM) in a Milestone Ethos Plus microwave digestion system (Milestone Srl, Sorisole, Italy). For the 3-week old rats, both pairs of tibias and both pairs of femurs (from the right and left back limbs) were utilised due to their small size; i.e. for each rat, the right and left tibias were digested together as were the right and left femurs, to obtain enough sample for analysis. Sample blanks were similarly prepared but without bone. The digested samples and sample blanks were transferred to 13 ml polypropylene tubes for ICP-OES analysis. The digested samples were analysed for Si using sample-based standards prepared by spiking aliquots of a pooled sample of the digests (1 mL was removed from each of the digested samples and pooled together) with Si (+ 0–2 mg/L) from a 100 mg/L multi-element standard. Silicon levels in the sample blanks was determined with Si standards (+ 0–10 mg/L) prepared in the 10% nitric acid solution. Silicon concentration of the bone samples was calculated using the method described below. Mean Si concentration of the sample blanks was 39.9 ± 2.8 μg/L (n = 16).

#### Skin samples

The skin samples (0.43–0.56 g) were digested as described above with 6–10 ml 10% nitric acid at 185 °C in the Milestone Ethos Plus microwave digestion system. The skin samples included some fur as it was not possible to remove it completely. The digested samples were analysed for Si with sample-based standards prepared by pooling 1 ml of the digested samples together and then spiking aliquots with Si (+ 0–2 mg/L) using a 100 mg/L multi-element standard. Sample blanks were similarly prepared but without skin samples and analysed with standards (+ 0–2 mg/L) prepared in 10% nitric acid. Silicon concentration of the skin samples was calculated using the method described below. Mean Si concentration of the sample blanks was 40.7 ± 1.1 μg/L (n = 8).

#### Feed and drinking water

The Si contents of the rat feed pellets and drinking water were also determined. Samples (0.15–0.30 g; n = 6) of the rat feed were digested in 5 mL 69% nitric acid at 180 °C (10 min ramp to 180 °C and then maintained at 180 °C for 20 min) in the Milestone Ethos Plus microwave digestion system. Four sample blanks were similarly prepared but without samples. Upon cooling the digested samples and sample blanks were diluted with 10 mL UHP water and transferred into pre-weighed 25 mL polypropylene tubes. The digested samples of feed, sample blanks and drinking water were analysed as a single batch for Si with multi-element standards (+ 0–20 mg/L) prepared in 2.5% nitric acid. Silicon concentration of the drinking water was obtained directly from the ICP-OES measurement, whilst the Si content of the feed was calculated using the method described below. Mean Si concentration of the sample blanks was 28.1 ± 0.1 μg/L (n = 4).

#### Calculation of tissue Si concentrations

Silicon concentration (μg/g) of the tissue samples and feed samples was calculated by the following method. Firstly, the Si concentration of the tissue or feed digest was blank corrected by subtracting the mean Si concentration of the appropriate sample blanks from the levels in the sample digest. Secondly, knowing the total volume of sample digest, the Si content (μg) of the digest was then calculated. Finally, the Si content of the digest was divided by the mass of the sample digested to obtain the Si concentration of the tissue sample. Silicon concentration of the tissue samples were expressed as wet weight for two main reasons. Firstly, this would allow direct comparison with previous studies [25,27] that reported Si levels in connective tissues (as per gramme wet weight of tissue) and secondly, it avoids (i) the pitfalls in drying tissues consistently and without introducing contaminant Si and (ii) the potential errors in handling the very low mass of dried tissues.

### Body silicon content

Silicon concentrations of the rat tissues were used to calculate/estimate the body Si content of the rat at the different ages for the tissues assessed. This was not a primary aim of the study and this calculation only came about during preparation of the manuscript. The total (whole) organ/tissue was collected for many of the samples analysed here and thus their mass (at the different ages) were known but, for some, where only a small representative sample of the tissue was collected (i.e. skin, bone, serum and vascular tissue), the total mass of the organ/tissue was obtained from published values for the female Sprague–Dawley rat at the different ages [35–42]. Silicon concentrations are not known or reported for adipose tissue, brain, gastrointestinal tract (organs + contents) or muscle and these were not recorded here. These account for, respectively, 7%, 0.6%, 7.7% (2.7% + 5%) and 40% of the adult rat's body mass with the remainder being 5.7% from, ‘rest of rat's body’ (i.e. miscellaneous; ref. [35] & Supplemental Table 2). With the possible exception of the gastrointestinal tissues, the Si contents of these tissues are expected to be low: the mass of muscle may make it a significant contributor overall, although previous reported values for Si are very low [25]. The gastrointestinal tract may have variable levels depending upon (i) recent absorption and (ii) presence or absence of silicate particles [43], although these are not contributors to the steady state body pool of Si and should be ignored for that purpose.

### Serum CTx

Serum CTx (carboxy terminal telopeptide of type I collagen) concentrations were obtained from our previous study using rats of the same strain and gender, i.e. female Sprague–Dawley rats [16]. In this published study, three groups of 3-week old rats (n = 40 rats in total) were maintained on the same diets, containing different levels of Si, for 26 weeks and monthly fasting blood samples were collected from five animals in each group [16]. Serum CTx was measured in the separated sera by commercial ELISA (RatLaps; Nordic Bioscience Diagnostics A/S). Serum CTx concentrations of the rats in the reference group, that were maintained on the standard rodent stock (maintenance) feed, was used here to compare with fasting serum Si concentrations with age. Apart from the different maintenance feeds, there was no other major difference between the two studies, making comparison of data between the two studies justifiable.

### Statistical analyses

Results are reported as means ± SD unless otherwise stated. Regression analysis (Curve Fit) was used to assess a change in Si concentration with age: i.e. higher concentration in young rats compared to adults or vice versa. Comparison of individual age groups was with One-way ANOVA, with Bonferroni post-hoc analysis to correct for multiple comparisons. A p value < 0.05 was considered significant. Pearson correlation (with 2-tailed *T*-test) was used to test for correlations between fasting serum Si and tissue Si concentrations, and between tibia and femur Si concentrations. All statistical analysis was conducted in IBM SPSS version 21 (IBM Corporation).

## Results

### Silicon content of the rat feed and drinking water samples

The maintenance diet contained 628 ± 66 μg Si/g feed and the drinking water Si concentration was 3.9 ± 0.07 μg Si/mL. These levels are typical of those previously found in rodent diets [16,44].

### Tissue silicon concentrations

**Fig. 1:**
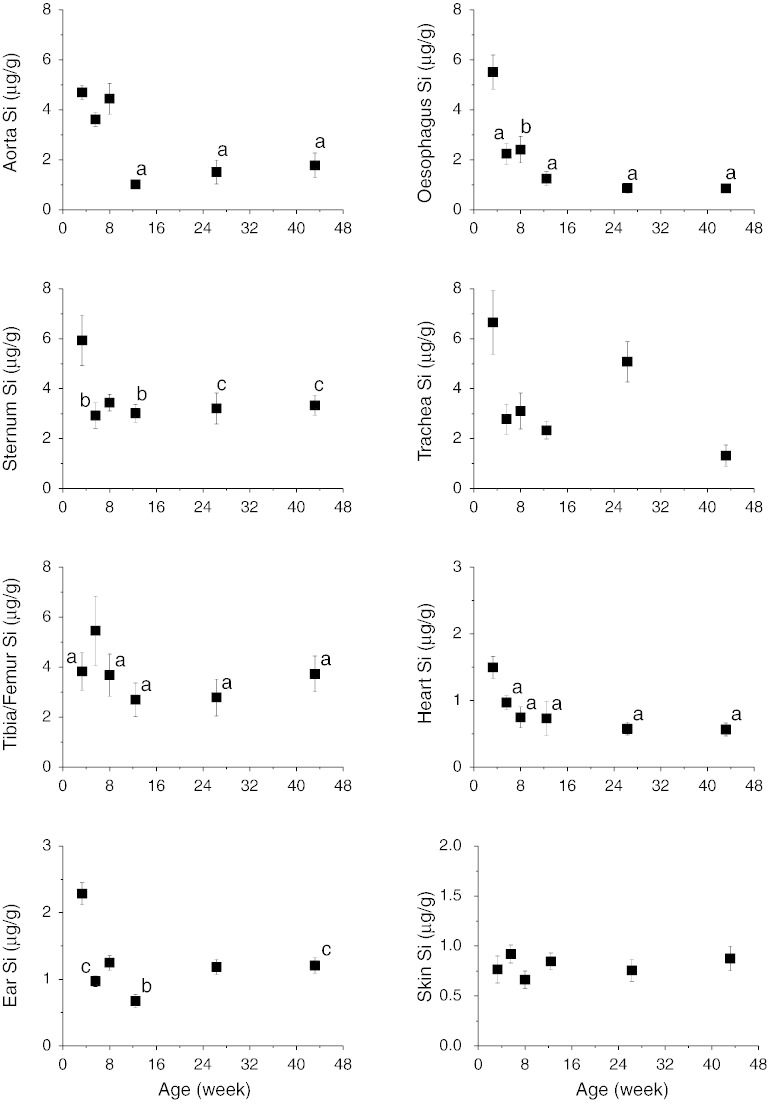
Silicon concentration (μg Si/g wet weight of tissue) of the connective tissues of female Sprague–Dawley rats of increasing age (3 weeks to 43 weeks). Data shown are means ± SE of 8–10 rats per age group. Bone Si values represent the mean of the femur and tibia bones (i.e. n = 16–20 bone samples per age group). ^a^p ≤ 0.0001, ^b^p ≤ 0.01, ^c^p < 0.05 compared to 3 week old rats except for bone where comparison was with 5 week old rats (One Way ANOVA with Bonferroni post-hoc analysis). Ear Si levels refer to the levels in the collagenous ear lobes.

**Fig. 2:**
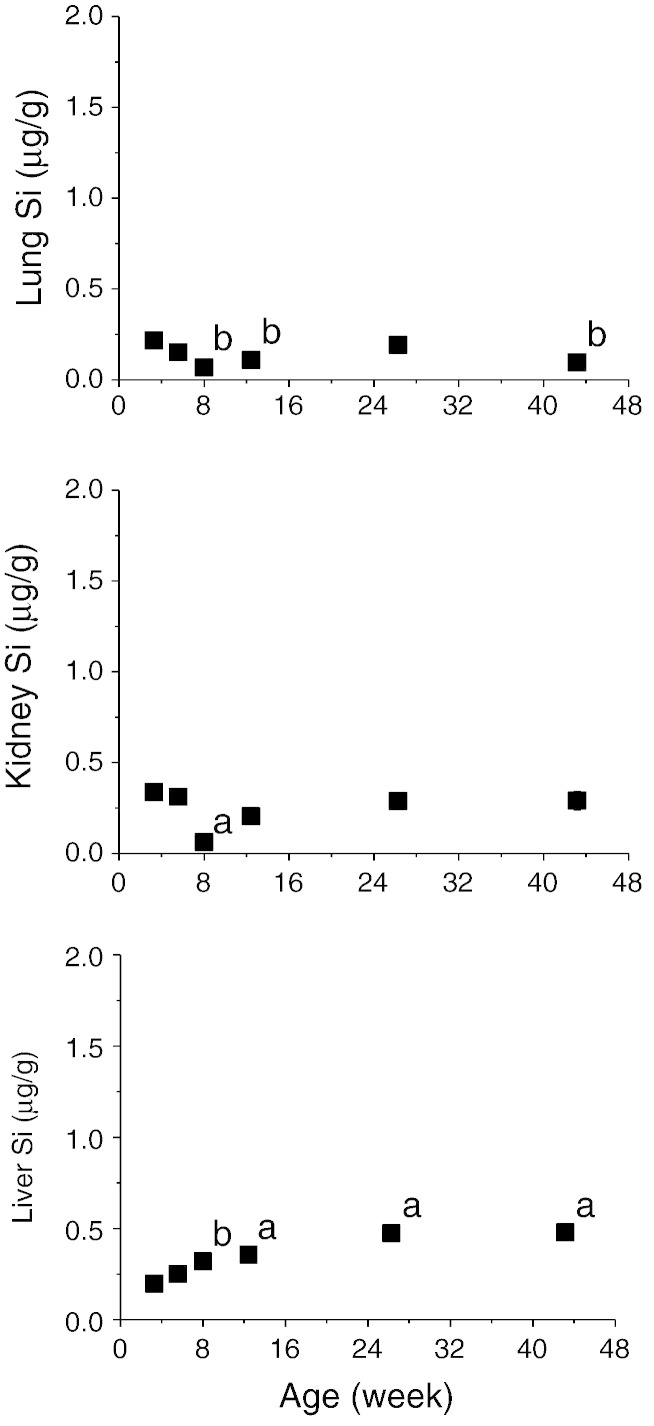
Silicon concentration (μg Si/g wet weight of tissue) of the non-connective soft tissues of female Sprague–Dawley rats of increasing age (3 weeks to 43 weeks). Data shown are means ± SE of 8–10 rats. ^a^p ≤ 0.0001, ^b^p ≤ 0.01, compared to 3 week old rats (One Way ANOVA with Bonferroni post-hoc analysis).

Silicon concentrations of the rat tissues (per gramme wet weight) are shown in Figs. 1 and 2. Tibia and femur Si concentrations were almost identical over the age range (Pearson correlation: r = 0.74, p < 0.0001) and hence average concentration of the two bones were used (Fig. 1). Higher Si concentrations (up to 50-fold, depending on age) were found in the connective tissues (Fig. 1 & Supplemental Table 1) compared to the non-connective tissues (Fig. 2 & Supplemental Table 1) and highest concentrations (up to 9.98 μg/g) were found in the 3 or 5 week old rats. Connective tissue Si concentrations then decreased by 2 to 6 fold with age, except the skin where it appears to be independent of age (p = 1). Regression analysis showed logarithmic associations between connective tissue Si concentrations and age (F > 16, p < 0.0002). No marked changes in Si concentrations were seen in the non-connective tissues, apart from the liver where it appeared to marginally increase by 2 fold with age (F = 130, p < 0.0001, regression analysis), albeit overall levels are low (< 0.5 μg/g; Fig. 2), compared to connective tissues. The decrease in Si concentrations (or the increase in the case of liver) occurred pre-puberty (i.e. < 12 weeks) and appeared to stabilise post-puberty, coinciding with the attainment of adulthood.

**Fig. 3:**
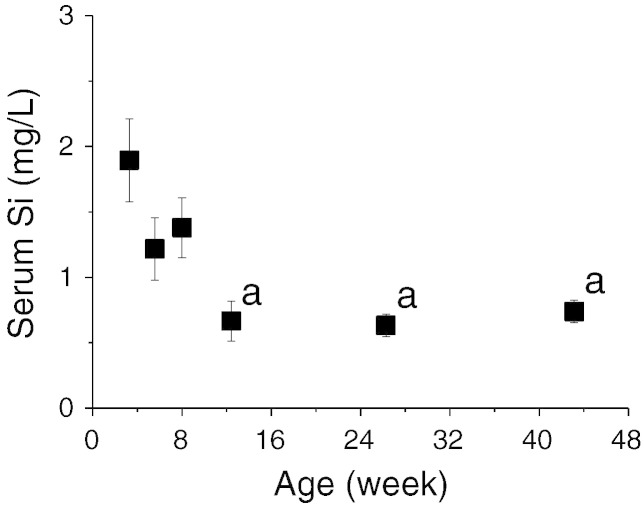
Fasting serum silicon concentration (mg/L) of female Sprague–Dawley rats of increasing age (3 weeks to 43 weeks). Data shown are means ± SE of 8–10 rats per age group. ^a^p ≤ 0.001, compared to 3 week old rats (One Way ANOVA with Bonferroni post-hoc analysis).

Circulating fasting serum Si concentrations (Fig. 3 & Supplemental Table 1) mirrored the changes (pattern) seen in the connective tissues, with highest Si concentrations in the 3-week old rats. Regression analysis showed significant logarithmic association between serum Si concentrations and age (F = 22.5, p < 0.0001). Significant correlations were obtained between fasting serum Si concentrations and Si concentrations of the connective tissues (Table 2).

### Total tissue silicon contents

**Fig. 4:**
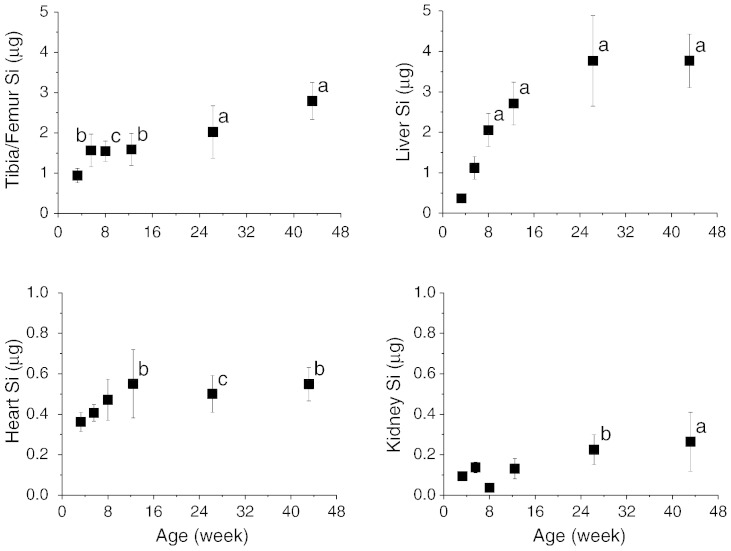
Comparison of the Si content (μg) of two connective and two non-connective tissues of female Sprague–Dawley rats of different ages (3 weeks to 43 weeks). Data shown are means ± SE of 8–10 rats. ^a^p ≤ 0.0001, ^b^p ≤ 0.01, ^c^p < 0.05, compared to 3 week old rats (One Way ANOVA with Bonferroni post-hoc analysis).

Taking into account the different size (mass) of the tissues, we compared the Si *contents* of two connective and two non-connective tissues at the different ages (Fig. 4). In all four tissues, an increase in *total* Si content was observed with age. In other words, whilst tissue Si concentrations falls with age (excluding the liver), the increase in organ size with growth means that absolute Si content (mass) actually increases with age (Fig. 4). However, the extent of the increase differed markedly between tissues. The liver showed the greatest increase, being 10-fold from 3 to 26 weeks, whilst the heart showed the lowest increase of 1.5-fold. Bone (i.e. tibia/femur) and kidney showed similar increases of 3-fold and the lack of tail-off suggested that further increases in Si content in these tissues could be expected with continued ageing. Regression analysis of tissue Si content showed a logarithmic association for liver (F = 201, p < 0.0001), and heart (F = 19.9, p < 0.0001) and a linear association for bone (F = 110, p < 0.0001) and kidney (F = 36.6, p < 0.0001) with age.

### Body silicon content

**Fig. 5:**
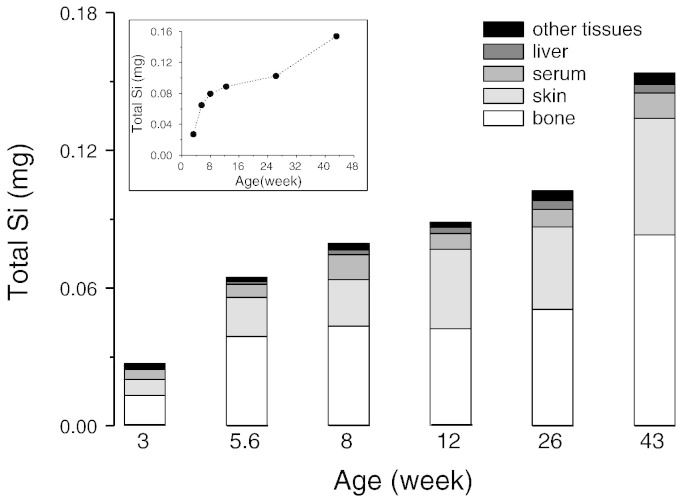
Summary of the measured tissue Si contents of female rats at different ages. The Si content shown accounts for tissues that make-up 36–39% of the rat's body mass. ‘Other tissues’ = the sum of: spleen, ear lobes, heart, trachea, oesophagus, aorta, lungs and kidneys. Silicon levels were not measured, and therefore not shown, for the remaining tissues that make-up the rest of the rat's body weight, namely muscle at 40%, adipose tissue at 7%, brain at 0.6%, gastrointestinal organs at 2.7% and miscellaneous (rest of rat body) at 5.7% (Supplemental Table 2). Silicon level in muscle tissue has previously been reported for 4-month (16-week) old male rats [25] and since muscle is the largest contributor to body mass, this reported value was used (after adjustment for relative differences in Si values between studies; see Results) to estimate how much Si muscle could contribute to total body Si content of the rat. Silicon content in muscle tissue was found to contribute an additional 64.8 μg (or 42%) and 75.3 μg (42%) to the total body Si content of the 12 and 26-week old female rats, respectively. However, bone and skin are still the largest contributors to total body Si content of the 12 and 26 week old rats, at 50.1 and 48.7%, respectively. Gastrointestinal contents account for 5% of total body weight (Supplemental Table 2), but are not relevant to systemic (body) Si levels. Insert graph shows the measured Si content as a function of age with the x-axis as a linear scale.

Body Si content for the tissues analysed is shown in Fig. 5 and Supplemental Table 3. Bone and skin accounted for 75–87% of the Si content measured, across the age range and, collectively, connective tissues accounted for 80–89% of the measured Si content. The analysed tissues however only account for ~ 36% of the rat's body mass (Supplemental Tables 2 & 3). Muscle and other tissues were not analysed but are expected to be low in Si. Carlisle [25], reported on the Si content of muscle tissue from 4 months (16 weeks) old male rats (specie not specified) and since muscle accounts for a large portion (~ 40%) of rat's body mass we estimated how much additional Si it could contribute to the total body Si content of the rat. In general, tissue Si values reported by Carlisle were higher than ours, so we first calculated the relative fold difference between our values, from the 12-week and 26-week old female rats (since we did not have 16-week old rats), and those reported by Carlisle [25] for skin, heart and liver (tissues with similar Si concentration to muscle) and then applied this adjustment (mean relative fold difference of 4.3 for both the 12 and 26 week old rats) to the muscle Si value reported by Carlisle to obtained an estimated Si concentration for muscle of 0.754 μg/g wet weight and 0.753 μg/g wet weight, respectively for the 12 and 26-week old rats. Taking into account that muscle contributes 40% to the rat's body weight, we estimated that Si from the rat muscle tissue would contribute an additional 64.8 μg (42%) and 75.3 μg (42%) Si to the total body Si content of the 12 and 26-week old rats, respectively. However, bone and skin are still the largest contributors to total body Si content of the 12 and 26 week old rats, at 50.1 and 48.7%, respectively.

### Correlations with collagen turnover

**Fig. 6:**
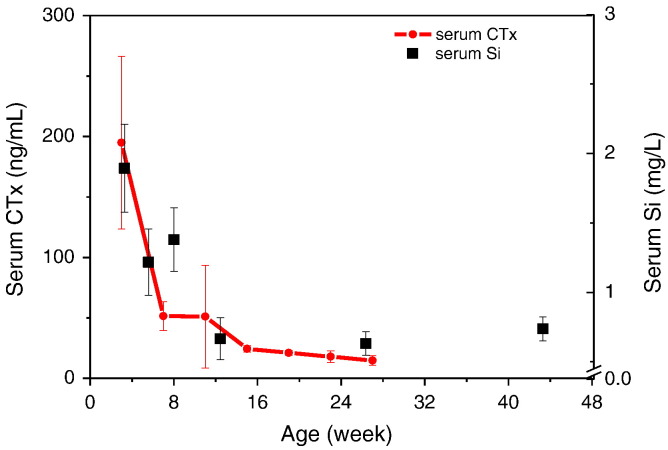
Mean type I collagen turnover, measured as C-terminal telopeptide of type I collagen (CTx) in fasting serum of female Sprague–Dawley rats of different ages (n = 5 rats per age group; ref. [16]) compared with fasting serum Si levels measured in this study (n = 8–10 rats per age group).

Serum CTx, a marker of type I collagen turnover, collected from a previous study [16], more or less mirrored the changes in serum Si concentrations with age (Fig. 6).

## Discussion

In this prospective, longitudinal and carefully controlled study we have confirmed the original proposals of (a) Carlisle [25], (b) Loeper et al. [27] and (c) Jugdaohsingh et al. [32], namely that (a) Si is associated with connective tissues, (b) connective tissue Si concentrations decrease with age and (c) that fasting serum Si is a good marker of the body pool of Si (i.e. connective tissue Si concentrations). However, a number of other important and novel findings were also obtained in this work and these are discussed below.

(1) Total Si concentrations of all tissues, including the connective tissues, are lower than previously reported [25], although the 4–5 fold proposed difference in Si concentration, between the connective and non-connective tissues of adult rats, was observed here. Difference in absolute levels is most likely due to improvements with modern methodology, for the preparation and analysis of Si that has occurred over the last 30–40 years. However, in the case of bone there is some evidence that differences in diet composition may influence the loading of Si into bone mineral [16,45] and thus affect total bone Si concentration. There is no evidence that this is similarly true for other connective tissues [44], but it remains possible that both differing environments (e.g. dietary Si exposure) and analytical methodology contribute to differences in historical and contemporary measures of tissue Si levels. The lack of methodological detail in the previous studies makes this difficult/impossible to resolve.
(2) Although there is no *unequivocal* evidence that mammals have a requirement for Si, estimation of total body Si content suggests that absolute Si requirements may be substantially lower than previously assumed, including for humans [46]. This was not, however, a primary objective of this study. When the total Si concentrations, acquired here, were used to estimate tissue Si contents and then whole body Si content of the rat, the amount of Si that may be required during growth/development was much lower than might have previously been assumed (Fig. 5), even if the estimated Si contribution from muscle tissue is included. Indeed, the difference in total body Si content between weanling (3-week old) and adult (26 week old) rats is less than 100 μg and 50% of this is acquired within the first 13 days after weaning. This (potential) very low requirement for Si may explain why so many Si deficiency studies, since Carlisle [4] and Schwarz & Milne [5], have failed to achieve frank Si deficiency, including the most comprehensive study to date where an extremely low dietary Si level was achieved (2–3 μg Si/g diet) albeit clearly not low enough [16]. It is possible, however, that in the studies of Carlisle [4] and Schwarz & Milne [5] the animals did not receive an optimal diet for growth and development and thus the addition of Si may have had a pharmacological effect.
  When the rat tissue Si values are extrapolated to an adult human based upon relative weights, then total body Si content amounts to only ~ 30 mg (or ~ 49 mg when the estimated Si contribution from muscle tissue is included), very much less that the 1–2 g that has been suggested to be present in the average human adult [47]. This may imply that Si requirement in humans is lower (i.e. 1–2 mg or less) than previously assumed (10–25 mg Si/d based on 24 h urinary Si excretion; 46) and may explain why a Si deficiency state or condition (disorder) has not been reported for humans. The ubiquitous presence of Si in our diet and in our environment makes it inconceivable for humans to become chronically deficient in Si. This finding of a lower requirement for Si is in agreement with a recent Si balance study in humans, showing that urinary and faecal Si excretions over 24 h can fully account for the total Si consumed (24 mg Si/d) in individuals who are in ‘Si balance' [48]. Although this study gave no indication whether any of the absorbed and urinary excreted Si had undergone exchange with the body Si pool, it is unlikely to be a high proportion [48]. Analysis of adult human tissues (bone, skin, liver, muscle and serum at the very least) will give a more accurate indication of Si requirement in adult humans. We expect it to be lower or not different to the above estimate. Silicon requirements in children are probably much higher (see below).
  This proposal of a lower requirement for Si in humans appears not to fit with our previous observational data reporting higher intakes of dietary Si to be associated with higher bone mineral density (BMD, a measure of bone strength) and thus better bone health [18,19]. We can provide two possible explanations for this apparent discrepancy. One possibility is that our estimate of total body Si content for humans is incorrect. Humans are not the same as rats and thus it’s possible that rat tissue Si levels are not representative of the levels in humans. Hence, adult humans may have a higher requirement for Si than adult rats and thus higher intakes of dietary Si provides optimal Si intake with regards to bone health. Another possible and, we believe, more likely explanation is that Si has two roles. Silicon may have an ‘essential’ role in connective tissues with regards to collagen health, and the amount of Si required for this role is minimal (small), as recently indicated from the direct quantification of Si in the collagen fraction of the rat bone [45]. This small amount of Si (associated with collagen) appears to be conserved in rats under different level of dietary Si exposure [45]. Under chronic dietary Si deprivation, we have previously reported that rats conserve their tissue Si levels by reducing their urinary output of Si [16]. Silicon may also have a second role, a ‘pharmacological’ role, in bone at least. This occurs through the incorporation of Si into the mineral fraction of bone, improving trabecular bone BMD and other bone quality measures [44]. This pharmacological effect is mediated systemically and is influenced by circulating levels of estradiol [44]. This pharmacological effect would explain the association between dietary Si intakes and BMD in humans and also the lack of tail-off in this relationship at the upper quintile of Si intake (30–63 mg/day) for men and pre-menopausal women [18].
(3) Our data suggest that young growing rats may have proportionally higher requirements for dietary silicon than adult rats. The higher concentrations of Si in the fasting serum and connective tissues of young rats, and the similar reported findings in humans [27,31,32] suggest that young mammals may have a higher requirement for Si, for bone and connective tissue development (see below).
(4) Fourthly, fasting serum and connective tissue Si concentrations may be markers of collagen (connective tissue) turnover. Here, fasting serum Si and connective tissue Si concentrations mirrored type I collagen turnover with age and a similar good fit between bone Si and bone hydoxyproline (collagen) concentrations (Supplementary Fig. 1) suggests that both fasting serum Si and bone Si concentrations may be biomarkers of collagen (connective tissue) turnover. Certainly bone and connective tissue turnover is higher in young rats, starting prenatally (mid-gestation embryonic day 14), peaking between postnatal days 7–21 and then decreasing rapidly thereafter to a stable level by postnatal days 30–40 (Refs. [49–51]). Bone collagen content and mechanical strength of the collagen matrix increases until three months of age in rabbits, when it attains maturity and prior to overall skeletal maturity at around 6 months [52,53].

The above findings deserved further investigation in studies that include younger rats for example (< 3 week old), as there is evidence from human studies that the peak of Si concentration may occur earlier and possibly in gestation [32]. In hindsight, muscle tissue and, probably also, adipose tissue, brain, and gastrointestinal organs should be collected and analysed for Si content to obtain a more accurate estimate of whole body Si level in the rat. Collectively the above findings also provide further evidence that Si is involved in bone and connective tissue development. Exactly what its role is remains unclear, although involvement in collagen synthesis and/or stabilisation seems likely [3,24,25]. Our recent report showing localisation of Si in the collagen fraction of the rat bone at a molar ratio of 1:6 Si:collagen, independent of the animal's age, supports a structural role for Si, as a cross-link between collagen units [45]. The nature of this interaction, between Si and collagen, is not known but the ex vivo demonstration of complexation between Si(OH)_4_ and sugars and hydroxyl-amino acids [54–57], suggests this is a real possibility. Potential binding sites for Si in collagen and other biomolecules have been proposed [20].

Finally, an interesting finding of this study was that Si seems to accumulate in the liver with age. Total Si content (and Si concentration) of the rat liver increased markedly with age and in the adult rat (26 week old) the liver accounts for ~ 4% of total body Si content and a similar figure was estimated for the adult human male liver. It is known that the liver is involved in the metabolism of silica nanoparticles and silicone released from silicone-filled implants [58–60]. For example, following intravenous or intraperitoneal injection of silica nanoparticles, Si accumulates in the liver and spleen via physical sequestered by macrophages, and this has been well characterised [59,60]. However, little is known/reported about the accumulation of dietary (or oral) silica in the liver. Dietary silicon is mainly in the form of soluble silica [Si(OH)_4_] or amorphous polymeric silica (phytolithic silica), and the latter, from the cereal (plant) component of the diet, requires breakdown in the gastrointestinal tract prior to absorption as Si(OH)_4_, a soluble and neutrally charged specie [3,61]. Thus it is unclear why this freely diffusible molecule should accumulate in or have an affinity for the liver. A previous study may provide some clues. Mehard & Volcani [62] reported that after injection of radiolabelled Si (^31^Si(OH)_4_), ^31^Si was taken up in the tissues, including the liver, but was subsequently rapidly released (eliminated), although a small percentage was bound or polymerised by liver cell organelles. A similar fate may thus exist for absorbed Si(OH)_4_. An alternate proposal is that some of the absorbed silica from the rat diet was in the form of nanoparticulate silica and it is this that gets sequestered in the liver.

## Disclosures

All authors declare no conflict of interest.

## Author's contributions

Authors' contributions: Study design: RJ and JJP. Study conduct: RJ, LDP and AIEW. Study oversight: RJ. Data analysis: RJ and LDP. Drafting manuscript & Revising manuscript content: RJ and JJP. Approving final version of manuscript: RJ, AIEW, LDP and JJP. RJ takes responsibility for the integrity of the data analysis.

## Figures and Tables

**Table 1:** Ages, numbers and life stages of the female Sprague–Dawley rats.

Rat age			Life stage
(days)	(weeks)	n	
23	3.3	10	Weanling
39	5.6	8	Puberty^a^
56	8	8	Sexual maturity^a^
87	12.4	10	
184	26.3	10	Social maturity
302	43.1	10	

a Rats are sexually mature between 6–8 weeks.

**Table 2:** Association between serum Si and tissue Si levels.

	Pearson correlation coefficients^a^	
Serum Si vs.	r	p
Aorta Si	0.51	0.00005
Heart Si	0.52	0.00004
Oesophagus Si	0.47	0.0008
Femur Si	0.42	0.001
Tibia Si	0.30	0.020
Lung Si	0.34	0.011
Liver Si	− 0.46	0.0004

a Pearson correlation (with 2-Tailed *T*-test).

## Appendix A Supplementary data

Supplementary materials (Supplementary Data, Supplemental Tables 1, 2 & 3 and Supplementary Figure 1) are available online.
