# Supplementary material for: The decrease in silicon concentration of the connective tissues with age in rats is a marker of connective tissue turnover
Source: Bone. 2015 Jun;75:40–8. doi: 10.1016/j.bone.2015.02.004 (PMC4406186; doi:10.1016/j.bone.2015.02.004)
Supplement: Supplementary file 1 — Supplementary materials (Supplementary Data, Supplemental Tables 1, 2 & 3 and Supplementary Figure 1) are available online. [file mmc1.docx]

**Supplementary Data**

**Supplemental Table 1.** Silicon concentration (µg Si/g wet weight of tissue) of the tissues of female Sprague Dawley rats of increasing age (3 weeks to 43 weeks).

| Tissues | Rat age (week) | | | | | |
| --- | --- | --- | --- | --- | --- | --- |
| Si (µg/g) | 3.3 | 5.6 | 8 | 12.4 | 26.3 | 43.1 |
| Aorta | 4.70 (0.29) | 3.61 (0.28) | 4.44 (0.62) | 1.02 (0.17) | 1.51 (0.48) | 1.77 (0.50) |
| Oesophagus | 5.51 (0.68) | 2.24 (0.41) | 2.41 (0.53) | 1.25 (0.27) | 0.87 (0.20) | 0.86 (0.18) |
| Sternum | 9.98 (4.13) | 2.92 (0.51) | 3.44 (0.33) | 3.02 (0.36) | 3.21 (0.62) | 3.33 (0.39) |
| Trachea | 6.65 (1.28) | 2.78 (0.60) | 7.69 (4.63) | 5.69 (2.39) | 5.08 (0.81) | 2.76 (1.46) |
| Tibia/Femur | 3.84 (0.75) | 5.46 (1.39) | 3.68 (0.84) | 2.69 (0.67) | 2.78 (0.74) | 3.73 (0.72) |
| Heart | 1.50 (0.17) | 0.97 (0.11) | 0.75 (0.16) | 0.73 (0.25) | 0.58 (0.09) | 0.56 (0.10) |
| Ear | 2.28 (0.17) | 0.97 (0.08) | 1.25 (0.11) | 0.67 (0.10) | 1.71 (0.53) | 1.21 (0.12) |
| Skin | 0.77 (0.14) | 0.92 (0.09) | 0.66 (0.09) | 0.85 (0.08) | 0.76 (0.11) | 0.87 (0.12) |
| Lung | 0.22 (0.02) | 0.15 (0.02) | 0.07 (0.02) | 0.11 (0.02) | 0.19 (0.03) | 0.10 (0.02) |
| Kidney | 0.34 (0.02) | 0.31 (0.02) | 0.06 (0.02) | 0.20 (0.02) | 0.29 (0.03) | 0.29 (0.05) |
| Liver | 0.20 (0.01) | 0.25 (0.02) | 0.32 (0.02) | 0.36 (0.02) | 0.47 (0.03) | 0.48 (0.03) |
| Serum (mg/L) | 1.89 (0.32) | 1.22 (0.24) | 1.38 (0.23) | 0.67 (0.15) | 0.63 (0.09) | 0.74 (0.09) |

Data shown are means (± SE) of 8-10 rats per age group.

Bone Si values represent the mean of the femur and tibia bones (i.e. n=16-20 bone samples per age group).

**Supplemental Table 2.** Relative organ weights as percentage of body weight in rats

| Tissues | % body mass^1^ | % body mass^2^ | % body mass^4^ |
| --- | --- | --- | --- |
| Adipose tissue | 7 |  |  |
| Blood | 7.4 | 7.82^3^ |  |
| Bones | 7.3 |  |  |
| Brain | 0.6 | 0.73 |  |
| GI contents | 5 |  |  |
| GI tract | 2.7 |  |  |
| Heart | 0.3 | 0.4 | 0.35 |
| Kidneys | 0.7 | 0.76 | 0.33 |
| Liver | 3.4 | 4.12 | 2.4 |
| Lungs | 0.7 | 0.71 | 0.51 |
| Muscle | 40.4 |  |  |
| Skin | 19 |  |  |
| Spleen | 0.2 |  | 0.17 |
| Rest of body | 5.7 |  |  |

^1^Male & female F-344 rats (35).

^2^Female Sprague Dawley rats, 271 g (35).

^3^Female Sprague Dawley rats, 244 g (37).

^4^Female Sprague Dawley rats, 350 g, 6-18 months rats (38).

**Supplemental Table 3.** Contribution of tissue silicon content (µg) to total silicon content in female Sprague Dawley rats of increasing age (3 weeks to 43 weeks).

|  | Rat age (week) | | | | | |
| --- | --- | --- | --- | --- | --- | --- |
| Si (µg) | 3.3 | 5.6 | 8 | 12.4 | 26.3 | 43.1 |
| Bone | 13.3 | 38.8 | 43.4 | 42.3 | 50.8 | 83.2 |
| Skin | 6.91 | 17.0 | 20.3 | 34.5 | 35.9 | 50.8 |
| Serum | 4.39 | 5.79 | 10.9 | 6.99 | 7.71 | 11.0 |
| Liver | 0.368 | 1.12 | 2.06 | 2.71 | 3.76 | 3.77 |
| Ears | 0.388 | 0.210 | 0.338 | 0.170 | 0.567 | 0.441 |
| Heart | 0.362 | 0.406 | 0.471 | 0.550 | 0.501 | 0.549 |
| Trachea | 0.313 | 0.184 | 0.807 | 0.461 | 0.444 | 0.684 |
| Sternum | 0.192 | 0.070 | 0.139 | 0.168 | 0.176 | 0.249 |
| Oesophagus | 0.141 | 0.092 | 0.099 | 0.067 | 0.050 | 0.070 |
| Aorta | 0.072 | 0.114 | 0.233 | 0.071 | 0.101 | 0.146 |
| Lungs | 0.347 | 0.427 | 0.380 | 0.431 | 0.567 | 0.488 |
| Kidneys | 0.187 | 0.274 | 0.073 | 0.262 | 0.450 | 0.528 |
| Total Si (µg) | 27.0 | 64.5 | 79.2 | 88.7 | 101 | 152 |

The Si content shown accounts for tissues that make-up 36-39% of the rat’s body mass. Silicon levels were not available for muscle at 40%, adipose tissue at 7%, brain at 0.6%, gastrointestinal organs at 2.7% and miscellaneous (rest of rat body) at 5.7%.

**Bone collagen concentration**

Materials

Ethylenediaminetetraacetic acid (EDTA, disodium salt dihydrate, 99% purity), *trans*-4-hydroxy-L-proline, bovine skin gelatine powder, isopropanol (99%, molecular biology grade), chloramine-T trihydrate (99%, ACS grade) and hydrochloric acid (37%, ACS reagent) were all from Sigma Aldrich Chemical Co (Gillingham, UK). Erlich’s Reagent (4-DABA, > 99% purity) was from Fluka Ltd (Gillingham, UK).

Method

Collagen concentration of the rat tibias was determined by extracting and measuring the hydroxyproline (HYP) concentration of the acid hydrolysed decalcified bones (63). Whole rat left tibias (n=4-5 per age group, except three week old rats where samples had been used for total Si analysis), were individually decalcified in dialysis bags (12-14 kDa) with 7.4 wt % saturated EDTA solution (37 g in 500 mL UHP water) over a two-week period at 4°C and then thoroughly washed and incubated in deionised water for a further two days. Samples (~ 8 mg) of the decalcified bones were freeze dried (LTE Mini Lyotrap, LTE Scientific Ltd, Oldham, UK) for 24 h in 0.3 mL crimp-top borosilicate glass vials (Chromacol, UK) and hydrolysed with vapour phase hydrochloric acid (HCl) in a CEM Discover Protein Hydrolysis system (CEM; Matthews, NC, USA). Samples were hydrolysed at 150°C for 45 min under anaerobic conditions (15 psi nitrogen). Vapour phase HCl was generated from 10 mL 6 M HCl added to the reaction vessel. The hydrolysed samples were reconstituted in 5 mL 50% isopropanol and 15 μL aliquots transferred to a 96-well microwell plate and mixed with buffered chloramine-T reagent for 5 min at room temperature. 0.15 mL of Erlich’s Reagent was then added and the chromophore allowed to develop for 30 min at 60°C before measurement of absorbance at 540 nm with an optical plate reader (Labsystems Multiskan RC). *Trans*-4-hydroxy-L-proline standards (0 to 0.25 mg/mL) were used to determine hydroxyproline content in the samples and bovine skin gelatine was used as a quality control.

**Supplementary Figure 1.**

**Supplementary Figure 1.** Comparison of bone hydroxyproline (HYP) concentrations (a marker of collagen concentration) with bone Si concentration of female Sprague Dawley rats of different ages (n=8-10 rats per age group). Note: data for the 3 week old rats are not shown as HYP concentrations was not collected for these samples. Hydroxyproline concentrations was determined in tibia bones (n = 4-5 per age group) decalcified with saturated solution (7.4 wt %) of ethylenediaminetetraacetic acid.
